# Religious/spiritual coping in people with chronic kidney disease: a cross-sectional study

**DOI:** 10.15649/cuidarte.2797

**Published:** 2024-05-01

**Authors:** Jéssica Sena-Tínel, Djenane Cristovam de Souza, Joice Requiäo Costa de Santana, Christielle Lidianne Alencar-Marinho

**Affiliations:** 1 Universidade do Estado da Bahia. Senhor do Bonfim- BA. E-mail: jessicatinel@hotmail.com Universidade do Estado da Bahia Universidade do Estado da Bahia Senhor do Bonfim- BA Brazil jessicatinel@hotmail.com; 2 Universidade Soberana. Petrolina-PE. E-mail: janacristovam@yahoo.com.br Universidade Soberana Petrolina-PE janacristovam@yahoo.com.br; 3 Universidade do Estado da Bahia. Senhor do Bonfim-BA. E-mail: jrequiao@uneb.br Universidade do Estado da Bahia Universidade do Estado da Bahia Senhor do Bonfim-BA Brazil jrequiao@uneb.br; 4 Universidade do Estado da Bahia. Senhor do Bonfim-BA. E-mail: christiellealencar@yahoo.com.br Senhor do Bonfim-BA christiellealencar@yahoo.com.br

**Keywords:** Renal Insufficiency, Chronic, Adaptation, Psychological, Nursing., Insuficiencia Renal Crónica, Adaptación Psicológica, Enfermería, Insuficiencia Renal Crónica, Adaptagáo Psicológica, Enfermagem

## Abstract

**Introduction:**

Chronic kidney disease (CKD) is considered a public health problem due to its high morbidity and mortality rates.

**Objective:**

To assess religious/spiritual coping in people with chronic kidney disease as a strategy for coping with the disease.

**Materials and Methods:**

This quantitative exploratory research was conducted out in a hemodialysis clinic. The sample consisted of 100 people with chronic kidney disease, undergoing hemodialysis treatment. The study used a socioeconomic demographic questionnaire and the Religious/Spiritual Coping Scale.

**Results:**

Altogether, 100 people participated in the study, with a mean age of 55.6 years and predominantly male (58%). Among religious/ spiritual coping variables (positive, negative, total), the mean value of total religious-spiritual coping was the highest. The means of positive and negative coping indicate that the former is more used. Patients whose religion was Candomblé had higher positive coping, whereas Evangelical patients had higher negative coping, and Spiritists had higher total coping.

**Discussion:**

Religious/spiritual coping in the study population was high, demonstrating its importance for coping with chronic kidney disease.

**Conclusion:**

The kidney patients who participated in this study were quite adept at using religious/spiritual coping expressively and positively, pointing out the importance of this practice in coping with kidney disease.

## Resumen

**Introducción:**

La enfermedad renal crónica (ERC) se considera un problema de salud pública debido a sus altos índices de morbimortalidad.

**Objetivo:**

Evaluar el afrontamiento religioso/ espiritual en personas con enfermedad renal crónica, como estrategia de afrontamiento de la enfermedad.

**Materiales y Métodos:**

Investigación exploratoria, con enfoque cuantitativo, realizada en una clínica de hemodiálisis. Los participantes fueron personas con enfermedad renal crónica, en tratamiento de hemodiálisis, siendo 100 participantes en total. Se utilizó un cuestionario demográfico socioeconómico y la Escala de Afrontamiento Religioso-Espiritual.

**Resultados:**

Participaron del estudio 100 personas, con una edad promedio de 55,6 años y predominantemente del sexo masculino (58%). Entre las variables de afrontamiento religioso-espiritual (positivas, negativas, totales), el valor medio del afrontamiento religioso-espiritual total fue el más alto. Entre los promedios de afrontamiento positivo y negativo, el primero parece ser el más utilizado. Los pacientes que tenían como religión el Candomblé mostraron un mayor afrontamiento positivo, el afrontamiento negativo fue mayor en los pacientes evangélicos y los espiritistas mostraron un afrontamiento total mayor.

**Discusión:**

El afrontamiento religioso-espiritual en la población estudiada fue alto, representando la importancia de esta práctica para el afrontamiento de la enfermedad renal crónica.

**Conclusión:**

Los pacientes renales que participaron en este estudio fueron muy hábiles en el uso del afrontamiento religioso-espiritual, de manera expresiva y positiva, señalando la importancia de esta práctica en el afrontamiento de la enfermedad renal.

## Resumo

**Introdujo:**

A doenga renal crónica (DRC), é considerada um problema de saúde pública, devido as suas taxas elevadas de morbimortalidade.

**Objetivo:**

Avaliar o coping religioso/ espiritual em pessoas com doenga renal crónica, enquanto estratégia de enfrentamento da doenga.

**Materiais e Métodos:**

Pesquisa exploratória, de abordagem quantitativa, realizado em uma clínica de hemodiálise. Os participantes foram pessoas com doenga renal crónica, em tratamento hemodialítico, sendo 100 participantes no total. Foi utilizado um questionário socioeconómico demográfico e a Escala de Coping Religioso Espiritual (CRE).

**Resultados:**

Participaram do estudo 100 pessoas, com média de idade de 55,6 anos e predominantemente do sexo masculino (58%). Entre variáveis do coping religioso-espiritual (positivo, negativo, total) a média dos valores do Coping Religioso Espiritual Total foi o mais alto. Já entre as médias do coping positivo e negativo, o primeiro se mostra mais utilizado. Pacientes que tinham o candomblé como religiáo, apresentaram maior coping positivo, O CRE negativo foi maior nos pacientes evangélicos e os espiritas apresentaram um CRE total maior.

**Discussao:**

O coping religioso espiritual na populagáo estudada foi elevado, representando a importancia desta prática para o enfrentamento da doenga renal crónica.

**Conclusao:**

Os pacientes renais que participaram desse estudo mostraram-se bastante adeptos ao uso do coping religioso espiritual, de forma expressiva e positiva, apontando a importancia dessa prática frente ao enfrentamento da doenga renal.

## Introduction

Chronic kidney disease (CKD) is considered a public health problem due to its high morbidity and mortality rates _^(1)^_. It is characterized by various changes that result in a progressive decrease in kidney function and has several causes and risk factors, including diabetes, high blood pressure, and behavioral and lifestyle factors _^(2)^_.

The total number of patients on chronic dialysis in Brazil in July 2021 was estimated at 155,781 _^(3)^_. Treatment is necessary to maintain these patients’ lives. However, it significantly affects their quality of life, the frequency of treatments, changes in routine, withdrawal from work activities, physical changes, and restrictions on water and food intake, which culminate in physical and emotional impacts^1^. All these factors can cause conflicts, including psychological ones, affecting spiritual skills, and interfering with coping with the disease^4^.

Hence, developing coping strategies is essential to carry on the treatment. Coping is a term that designates various cognitive and/or behavioral methods that individuals can use to face situations considered stressful^5^, with particular attention to those related to religiosity and/or spirituality^6^. American psychologist Kenneth Pargament was responsible for developing the theory on religious/ spiritual coping (RSC), defined as the use of religiosity and/or spirituality to face moments of difficulty and stress in one's life^7^. It is essential to evaluate RSC, as these strategies can be crucial in the treatment and management of these patients and a great ally for health professionals who monitor them^8^.

Fear and anxiety arise when dealing with life-threatening situations, so the individual turns to a supreme being, according to their beliefs, hoping to improve their health status. The search for religiosity/spirituality increases when the person faces difficulties such as financial ones, loss of health, or loss of people, making them realize the brevity of life, which leads people to search for meaning in life and/or emotional support^9^.

The relationship between religious/spiritual coping and CKD has been extensively studied, and research shows that spirituality and/or religiosity provide the ability to better cope with pain and treatment, decrease depressive symptoms, and strengthen hope^4^^,^^6^. Spirituality is related to the sacred and/or divine, and may or may not extend to a religion^10^. Religiosity is related to traditions, celebrations, and knowledge of sacred books, which is a partial manifestation of spirituality^1^.

Health professionals who care for these patients should understand the importance of addressing these coping measures and offering adequate support as they go through the disease^11^. Nursing professionals are the ones who have the most contact with these patients, so they must be treated holistically, encouraging involvement with religiosity and spirituality to better accept their pathology, and reduce the appearance of complications, such as mental ilnesess^12^.

Thus, this study aimed to assess religious/spiritual coping in people with CKD.

## Materials and Methods

This cross-sectional quantitative study was carried out in a hemodialysis clinic in a city in inland Bahia, Brazil. The study participants were CKD patients undergoing hemodialysis treatment. The eligibility criteria were patients diagnosed with CKD, over 18 years old, undergoing hemodialysis treatment for at least 6 months, able to communicate, and with no difficulty responding to the instruments. During the study period, there were 198 participants eligible to participate in the research. The number of participants was estimated based on the sample size calculation, considering a 95% significance level and a 5% margin of error, totaling 110 patients. However, data were collected from 100 patients through nonprobabilistic convenience sampling. The losses were due to social isolation because of the COVID-19 pandemic, which broke out during the study development.

Data were collected from July 2019 to February 2020. Patients were approached at the dialysis service, in a private location, to inform them about the research objectives and procedures and explain the informed consent form, which they signed.

The database was stored on Mendeley Data^13^. The questionnaires were typed into 2010 Microsoft Excel, with the coding variables to be analyzed. Data were processed on R 4.0.2 (statistical programming language software). Univariate analysis was performed to describe the profile of patients who participated in the study. Next, nonparametric statistical tests were applied to independent samples to verify whether there was significant evidence of association between the variables of interest.

The study used a socioeconomic and demographic questionnaire prepared by the authors with data on sex, age, marital status, number of children, education level, income, and religion. Data on coping were collected with the Religious/Spiritual Coping Scale.

The Religious/Spiritual Coping Scale was developed and validated in Brazil and aims to assess how individuals use religion/spirituality to deal with stressful situations. The instrument has 87 items, of which 66 are distributed across eight factors of the Positive Religious Spiritual Coping (PRSC) dimension, represented by P1, P2, P3, P4, P5, P6, P7, P8 (P1 - Transformation of self and/or of one’s life; P2 - Actions in search of spiritual help; P3 - Offering help to others; P4 - Positive attitude towards God; P5 - Personal search for spiritual growth; P6 - Actions in search of the institutional other; P7 - Personal search for spiritual knowledge; P8 - Distancing through God, religion, and/or spirituality). The other 21 items are distributed across four factors of the Negative Religious Spiritual Coping (NRSC) dimension, represented by N1, N2, N3, N4 (N1 - Negative reevaluation of God; N2 - Negative attitude towards God; N3 - Negative reevaluation of meaning; N4 - Dissatisfaction with the institutional other). The classification between positive and negative RSC strategies arises from the consequences they have for those who use them^14^.

The following parameters were used to analyze and interpret the RSC scores according to the participant’s use: none or negligible = 1.00 to 1.50; low = 1.51 to 2.50; average = 2.51 to 3.50; high = 3.51 to 4.50; very high = 4.51 to 5.00.

The data were transcribed into the 2013 Excel for Windows and then transferred to the Data Analysis and Statistical (STATA) software, version 14.0, to perform descriptive analyses and statistical inference, creating frequency tables and measures of position for sociodemographic data.

The positive, negative, and total coping variables were associated with religion and age. The Shapiro- Wilk test was used to check the normality of the variables. The ANOVA and Kruskal-Wallis tests were used for associations between coping variables and religion, and the Pearson Correlation and Spearman Correlation (rO) were used for the correlation between age and coping. The confidence interval was set at 95%, and p-values < 0.05 were set as significant.

This study followed the criteria according to Resolution no. 466/12 of the Brazilian National Health Council, which regulates human research, and was approved by the Research Ethics Committee of the State University of Bahia, according to evaluation report no. 3.374.401/2019.

## Results

The study sample comprised 100 people, with a mean age of 55.60 years, predominantly males (58.00%). There was a prevalence of low income, as 79% received up to one minimum wage, and 50.00% were married.

They had been on hemodialysis treatment for a mean of 5.9 years. Most participants were Catholics (66.00%), followed by Evangelicals (24.00%) (Table 1).

**Table 1:** Sociodemographic characteristics of patients with chronic renal disease on hemodialysis-Senhor do Bonfim, BA, Brazil, 2020 (n = 100)

Characteristics	n (%)	95% CI
Sex Males Females	58 (58.00) 42 (42.00)	47.90 - 67.40 32.50 - 52.00
Marital status Single Married Divorced Widow(er) Others	29 (29.00) 50 (50.00) 9 (9.00) 11 (11.00) 1 (1.00)	20.80 - 38.70 40.10 - 59.80 4.70 - 16.50 6.10 - 18.90 0.10 - 6.90
Income Up to 1 MW 2 to 3 MWs > 4 MWs No income	79 (79.00) 14 (14.00) 1 (1.00) 5 (5.00)	70.50 - 86.60 84.70 - 22.60 0.10 - 7.00 2.00 - 11.70
Religion Catholic Evangelical Spiritist Candomblé No religion	66 (66.00) 24 (24.00) 1 (1.00) 1 (1.00) 8 (8.00)	56.00- 74.70 16.50 - 33.40 0.10 - 6.90 0.10 - 6.90 4.20 - 19.20
Age Years of education Number of children. Median(II) Time of treatment (years). Median(II)	Mean 55.60 ± 13.67 5.50 ± 4.59 3 (1; 4) 4(1,5; 10)	52.80 - 58.30 4.57 - 6.40 2.40 -3.30 4.80 - 6.90

*95% CI: 95% confidence interval. II (Interquartile Range)*

The mean value of Total Religious Spiritual Coping (TRSC) was the highest (3.91) of its three variables (positive, negative, total). Also, PRSC was more used than NRSC, with a higher mean (3.06). Among the eight PRSC factors, P4 (Positive attitude towards God) had the highest mean (3.54) and factor P2 (Actions in search of spiritual help) had the lowest one (2.22). In NRSC, N2 (Negative attitude towards

God) stood out with the highest mean (2.76), while N1 (Negative reevaluation of God) had the lowest mean (1.44) (Table 2).

**Table 2:** Assessment of positive, negative, and total religious/spiritual coping (RSC) and patients' positive and negative RSC factors - Senhor do Bonfim, 2020 (n = 100)

	Mean ± SD	Interquartile range
Positive RSC	3.06 ± 0.80	3.05 (2.75 - 3.40)
Negative RSC	1.82 ± 0.50	1.76 (1.49 - 2.00)
Total RSC	3.91 ± 0.46	4.00 (3.62 - 4.24)
RSC dimensions		
P1 - Transformation of self and/or of one’s life	3.15 ± 0.70	3.25 (2.67 - 3.64)
P2 - Actions in search of spiritual help	2.22 ± 0.70	2.13 (1.75-2.50)
P3 - Offering help to others	3.25 ± 0.85	3.29 (2.86- 3.71)
P4 - Positive attitude towards God	3.54 ± 0.60	3.55 (3.27-3.91)
P5 - Personal search for spiritual growth	3.04 ± 0.90	3.00 (2.40- 3.80)
P6 - Actions in search of the institutional other	3.00 ± 0.80	2.90 (2.50-3.50)
P7 - Personal search for spiritual knowledge	2.43 ± 0.80	2.32 (1.80- 3.00)
P8 - Distancing through God, religion, and/or spirituality	3.49 ± 0.78	3.50 (3.00- 4.00)
N1 - Negative reevaluation of God	1.44 ± 0.54	1.25 (1.00-1.75)
N2 - Negative attitude towards God	2.76 ± 0.84	2.75 (2.25- 3.25)
N3 - Negative reevaluation of meaning	1.91 ± 0.85	1.80 (1.20- 2.30)
N4 - Dissatisfaction with the institutional other	1.51 ± 0.68	1.25 (1.00-1.75)

*CI: confidence interval; SD: standard deviation*

The results of the association between religions and PRSC, NRSC, and TRSC showed significant differences. Patients whose religion was Candomblé had higher PRSC (4.11), Evangelical ones had higher NRSC (2.04), and Spiritists had higher TRSC (Table 3). Moreover, significant correlations were found between age and NRSC and TRSC. Age was negatively correlated with NRSC (rO = -0.209) and positively correlated with TRSC (rO = +0.206) (Table 3).

**Table 3:** Association between coping and religion and correlation between coping and age of chronic kidney disease patients on hemodialysis - Senhor do Bonfim, BA, Brazil, 2020 (n = 100)

	PRSC	NRSC	TRSC
Mean p-value	Mean p-value	Mean p-value
Religion Catholic Evangelical Spiritist Candomblé Others No religion	0.014* 3.04 ± 0.51 3.24 ± 0.56 3.32 ± 0.00 4.11 ± 0.00 2.65 ± 0.79 2.32 ± 0.79	0.011† 1.75 ± 0.64 2.04 ± 0.10 1.19 ± 0.00 2.00 ± 0.00 1.97 ± 0.21 1.58 ± 0.14	0.0072† 3.98 ± 0.54 3.68 ± 0.92 4.62 ± 0.00 3.90 ± 0.00 3.80 ± 0.20 4.28 ± 0.12
Age. rO	0.0237 0.81‡	-0.209 0.03§	0.206 0.03§

*ANOVA tKruskal-Wallis tPearson Correlation §Spearman Correlation

*PRSC: Positive Religious Spiritual Coping NRSC: Negative Religious Spiritual Coping TRSC: Total Religious Spiritual Coping*

## Discussion

The predominance ofmales among CKD patients is commonlyfound in both national and international research. Most participants in this study were Catholics, which corroborates similar research carried out in a renal replacement therapy unit in inland Sao Paulo to assess hemodialysis patients’ attitudes toward pain and their relationship with spirituality^4^. This may be related to the fact that the Catholic religion is the most prevalent in this country.

The mean age was 55.60 years, and there was a prevalence of low education level and income, which was equivalent to up to one minimum wage. Also, 50% were married. These data are in line with a Brazilian study that investigated the association between religiosity/spirituality and happiness in patients undergoing hemodialysis - they were predominantly middle-aged adults (57.8%) and had a low education level, as 60.90% only finished middle school^10^.

The study population had high RSC, indicating the importance of this practice for coping with kidney disease. Data from a Brazilian study that sought to demonstrate the relationship between RSC and hope in patients undergoing chemotherapy treatment showed that all patients in the study used RSC as a coping strategy^15^. The greater the use of RSC, the greater the beneficial effects on patients' lives, highlighting improvements in general health, greater vitality, and increased hope^16^. Quality of life and spiritual well-being are better developed in those who rely on religion/spirituality^1^.

When evaluated separately, PRSC was higher than NRSC, which shows that patients use more of the positive strategy. This data is consistent with a study carried out in Minas Gerais, which evaluated the spiritual well-being and RSC of patients on hemodialysis, showing that the PRSC had a high mean score (3.34) and was used more than the NRSC^1^. Greater use of NRSC is expected to be more linked to feelings such as spiritual distress^17^. A Brazilian study carried out in Fortaleza with patients with chronic obstructive pulmonary disease shows a significant relationship between NRSC and depressive symptoms^10^.

Using coping to deal with the disease helps reduce anxiety because the belief that God, a greater being, is in control of the situation, acting in times of illness, brings a sense of relief. It can also be used to ease these patients’ pain symptoms, as it brings a feeling of comfort. RSC is also associated with improved quality of life and reduced depression in hemodialysis patients^16^.

The influence of spirituality and beliefs provides support when facing daily changes brought on by the disease. Spirituality brings a greater feeling of inner strength^18^. Among the PRSC and NRSC factors, the greatest positive highlight was P4 (Positive attitude towards God), in which one has God as a foundation, seeks Him, makes supplications, and works together with Him; they try to positively reassess their condition through God^14^. The belief in a superior being, who can control the external events of their life, helps the individual feel safer and reduces the focus on their pathology and the limitations it can bring^19^.

Factor N1 (Negative reevaluation of God) stood out in NRSC, which occurs when the individual begins to question the existence or love of God and believes that God is punishing them for some personal reason^14^. As they face suffering, the individual may feel abandoned by God and/or stop believing in his existence, thus experiencing a negative spirituality/religiosity^19^. This negative feeling is very common at the beginning of treatment, during the process of accepting the diagnosis. This period can be marked by doubts regarding religiosity and the transfer of responsibility for the disease to God, who is seen as the one who caused it^20^.

Age had a significant relationship with coping. The older the person, the more religiosity and spirituality they used to cope with stress during treatment. Older people use these mechanisms as a strategy to deal with adversities, such as loneliness, distance from family members, and physiological changes resulting from aging. Hence, the refuge of religiosity/spirituality brings a feeling of security and protection^21^. Furthermore, the level of spirituality is lower in the younger group, which may interfere with the use of coping^17^.

Candomblé was the religion with the highest PRSC, whereas NRSC was higher in Evangelical patients. A Brazilian study with 129 participants that investigated the relationship between spiritual anguish and RSC use showed that non-Catholic patients had greater use of NRSC^17^.

When a person believes in something greater, divine, even in an afterlife, and that their illness is only part of the journey, they tend to have greater acceptance of the illness and deal better with suffering^6^. A study carried out in Jordan with 218 patients undergoing hemodialysis treatment showed that participants who made greater use of spirituality were less likely to develop depression and anxiety. They used religion as a mechanism, aiming to achieve well-being and have greater comfort and security^22^.

Various studies highlight that the multidisciplinary healthcare team, especially nursing, must know about the influence of spirituality/religiosity on patients' lives. It is necessary to know the patients' experiences regarding this aspect and how each one understands the concepts, and how they use it to cope with the pathology and its treatment. Professionals must encourage these practices, which can bring a feeling of support, establishing a greater bond between professionals and patients^1^^,^^10^.

This is a rather significant health topic, especially in nursing, as they are generally the professionals who have the most contact with these patients. Understanding the influence that RSC has on the lives of these patients is crucial, knowing their needs, not just focusing on one aspect of these individuals' lives, but seeing it holistically, giving due importance to the psychological and spiritual aspects as well. Thus, further studies should address this topic, investigating the importance of religion, spirituality, and RSC in these living conditions, and also considering a qualitative approach, which can complement the results with the patient's perception of the topic. The cross-sectional design stands out as a limitation of the study, as it addressed a single moment and a single hemodialysis center, which does not allow extrapolation of the results.

## Conclusion

The kidney patients who participated in this study were quite adept at using RSC expressively and positively, pointing out the importance of this practice in coping with kidney disease. Age was positively correlated with PRSC and negatively correlated with NRSC. Candomblé was the religion with the highest PRSC, Spiritists had a higherTRSC, while NRSC was more used by Evangelical patients.

This topic has been increasingly studied, highlighting its importance in the face of various chronic pathologies, in which patients need numerous strategies to face their health conditions. Therefore, health professionals should know about this topic, its influence on better acceptance of chronic disease, and know how to approach it, helping patients to use it. Brazil is a quite religious country - hence, it is greatly important to study this topic. Further studies are needed to cover this subject, which can highlight the influence and impact that coping has on the lives of CKD patients and investigate health professionals’ experience on this subject.
